# Gynæcology and Obstetrics

**Published:** 1906-01-20

**Authors:** 


(*To be continued.*)

### GYNÆCOLOGY AND OBSTETRICS.

**Mortality during the Puerperium.**—The death-rate from puerperal septic diseases still remains terribly high. Whilst in London it has been steadily declining for the last decade or more, in other parts of the kingdom it is as high as ever, or even higher, according to some statistics collected by Boxall.[14] The statistics do not show whether the satisfactory decrease in London is general or only confined to the intern and extern practices of the various lying-in hospitals. In his annual report as Master of the Rotunda Hospital, Hastings Tweedy [15] points out that a low morbidity rate is a more accurate guide to the efficiency of a maternity institution than an actual mortality. The standard of morbidity employed for years, a rise of temperature to or above 100.8° F., Tweedy considers too high and arbitrary as a limit. On the Continent the standard is slightly lower—namely, 100.4° F. As a basis for treatment Tweedy has adopted as an indication of morbidity a temperature running above 99° F. for 36 hours, and accompanied by a pulse-rate of over 89 per minute. His method is to search carefully for any cause of the symptoms as they arise without waiting for the 36 hours' limit if these are acute. If the parturient canal is suspected a vaginal douche is given, a purgative administered, and the head of the bed raised on blocks to promote free drainage. If symptoms persist the following day the vagina is again douched, a Fergusson's speculum passed, the cervix wiped dry with sterile wool, and a suitable sterile glass tube with aspirating syringe attached passed into the uterus to aspirate its contents. On withdrawal this tube is immediately sealed and sent to the pathological department for bacteriological examination, and the uterus is douched with salt and water, peroxide of hydrogen, or cyllin solutions. After another 24 hours without improvement the patient is isolated, with her nurse, bedding, and belongings, and, if the bacteriological report is positive, the uterus is explored with the gloved forefinger for pieces of retained placenta, membranes, or blood-clot. The present master has entirely discontinued the practice of his predecessor, who employed the flushing curette combined with tight packing of the uterus with iodoform gauze. All the serious cases received stimulants, abundant nourishment, and large doses of perchloride of iron. Calomel and mercurial inunctions were also employed frequently, and one very severe case of pyæmia improved rapidly on the free inunction of Credé's ointment, collargol 17 grains to the oz. of lard. Of the 32 grave cases which occurred in the Rotunda eight died. But the majority of the morbid cases were slight, and recovered quickly without treatment or with one or more vaginal or intrauterine douches. Various changes have been introduced at the Rotunda for the prevention of sepsis. These include the use of rubber gloves and finger stalls by nurses, students, and officers; the use of forceps for washing morbid cases, to diminish the liability of the nurses to sore fingers; the numbering and complete isolation of each bed and its belongings; and the use by the patients of sterilised gamgee diapers. A separate bed pan, utensil, and face basin are now provided for each bed, and these are boiled after each patient is discharged—a procedure to which considerable importance is attached. The tow wipes are boiled daily, and kept steeping in lysol solution. Where manual removal of the placenta is necessary, Tweedy advocates the repeated introduction into the uterus of the gloved hand until every particle of adherent tissue has been removed, so that no septic focus may remain. He only employed antistreptococcic serum in one case, and with discouraging results. Success, however, sometimes follows its use. P. H. Ward [16] has employed it in a patient in whom excessive uterine tenderness before delivery suggested some antepartum infection. Small portions of placenta were retained, but the temperature remained high after these were removed. It fell rapidly, however, after a fourth injection of 10c.c. of polyvalent antistreptococcic serum. This patient had severe postpartum hæmorrhage, and symptoms of septicæmia set in on the evening of the third day. In another case successfully treated by W. Blackwood,[17] symptoms of pyæmia and pylephlebitis appeared on the fourth day. The child had been born, and the placenta expelled before the arrival of midwife or doctor, and infection was ascribed to dirty bedding. In the course of six days 80c.c. of antistreptococcic serum were administered. The temperature, however, did not become normal until a fortnight later, after several abscesses had been opened and drained. Sir Arthur Macan and Dr. Purefoy,[18] two past masters of the Rotunda, have both recently expressed their disappointment with the serum treatment. The latter is still a believer in the value of the flushing curette, while he disapproves of Tweedy's plan of "repeated introduction of the gloved hand into the uterus" for the removal of retained placenta, fearing the admission of air and the opening up of avenues of infection.

[14] Brit. Med. Jour., May 20, p. 1094. [15] Dublin Jour. Med. Sc., June. [16] Lancet, June 10. [17] Ibid., Oct. 28, p. 1253. [18] Ibid., June 17, p. 1650.

(*To be continued.*)

